# Early detection of urological malignancies in Lynch syndrome: a systematic review

**DOI:** 10.1007/s10689-026-00560-5

**Published:** 2026-04-24

**Authors:** B. H. J. Doornweerd, M. W. Rasmussen

**Affiliations:** 1 Department of Urology, University of Groningen, University Medical Center Groningen, Groningen, The Netherlands; 2 The Danish HNPCC Register, Gastro Unit, Copenhagen University Hospital - Amager and Hvidovre, Copenhagen, Denmark

**Keywords:** Lynch syndrome, Urothelial carcinoma, Hereditary cancer, Prostate cancer, Surveillance, Cancer prevention

## Abstract

Lynch syndrome predisposes to multiple cancer types, including urological malignancies. However, no evidence-based recommendations for surveillance of urological malignancies currently exist. In this systematic review, we aim to describe the current evidence regarding surveillance for these cancers. A systematic literature search was conducted using MEDLINE, searching for urological malignancies, Lynch syndrome, and surveillance including the results of the surveillance methods. Sensitivity and specificity were calculated, when possible, preferably for pooled data from each surveillance method. The risk of bias was assessed using the Newcastle–Ottawa Scale. After full text-screening, nine studies published in 2008–2025 met the inclusion criteria, including two on prostate cancer and seven on urothelial cancer. Prostate cancer-antigen (PSA) surveillance led to 38 prostate cancer diagnoses in 865 individuals with Lynch syndrome, with 71% being clinically significant prostate cancers. In 1564 individuals, 11 urothelial carcinomas were diagnosed by different surveillance methods and 15 diagnoses were missed. Sensitivity of urinalysis, urine cytology, urine MSI, and CT and cystoscopy was 11%, 30%, 100%, and 100% respectively. Specificity was 90%, 97%, 99%, and 100% respectively for these methods. Further data is needed but for prostate cancer surveillance PSA shows promise, while for *MSH2* carriers at least, urine microsatellite instability analysis shows promise as a urothelial cancer surveillance test.

**Supplementary Information:**

The online version contains supplementary material available at 10.1007/s10689-026-00560-5.

## Introduction

Lynch syndrome (LS) is a hereditary cancer predisposition syndrome caused by pathogenic variants in the mismatch repair (MMR) genes *MLH1*, *MSH2* (including epigenetic silencing through *EPCAM*), *MSH6*, and *PMS2* [1]. Individuals with LS are at increased risk of developing cancer at a younger age than those with sporadic cancer. In addition to the well-established elevated risks for colorectal and endometrial cancer, LS is also associated with an increased risk of urological malignancies, including cancer of the prostate, ureter and kidney, and urinary bladder [2–5]. Little is known about the risk in LS for rare urological malignancies such as germ cell tumors of the testis or penile carcinoma. A small number of testicular cancers has been reported in a large LS database and an association of germ cell tumors with LS might exist, but testicular cancer is rarely the first cancer diagnosed in LS [6–8]. An increased incidence of renal cell carcinoma has been reported as well [9], but in general, renal cell carcinoma, germ cell tumors and penile cancer are not considered as being associated with LS.

Cumulative incidence for prostate cancer (PC) at 75 years is approximately 24% for male *MSH2* carriers [2] and clinically significant PC is more frequently observed in carriers of pathogenic *MSH2* variants compared with non-carriers [10]. Urothelial carcinoma (UC) of the upper urinary tract and bladder are common malignancies in *MSH2* carriers with a cumulative incidence at 75 years of approximately 18–19% and 8–13%, respectively [2]. While the most common malignant cause of death for LS individuals is colorectal carcinoma, both PC and UC are among common malignant causes of death in LS [11].

To improve treatment outcome and survival, asymptomatic individuals with LS are advised to undergo periodic surveillance for colorectal cancer, but there is no consensus on surveillance strategies for urological malignancies in LS [12]. In individuals with a family history of UC, UC surveillance is however recommended [13].

For PC surveillance, the most used test is prostate-specific antigen (PSA). Elevated PSA levels are usually followed by a multiparametric magnetic resonance imaging (MRI) of the prostate and/or prostate biopsies to confirm a diagnosis [14].

In contrast to PC, there is not a clearly defined surveillance test for UC. Several tests have been recommended, including urinalysis (to detect hematuria), urine cytology, and ultrasound of the abdomen [13]. More recently, urine-based microsatellite instability (MSI) analysis has been researched as a surveillance test in LS [15]. In high-risk LS individuals with a family history of UC, computed tomography (CT) and cystoscopy have also been used for surveillance [16], normally being the gold standard for the diagnosis of UC [17].

Although there is a multitude of recommendations on urological surveillance [18], there is a lack of evidence supporting these recommendations. This systematic review aims to summarize the currently available evidence for early detection of urological malignancies in LS by surveillance.

## Methods

This systematic review was conducted using the PRISMA 2020 checklist. A three-step approach was followed, consisting of relevance assessment, quantitative outcome extraction, and study quality evaluation. Study relevance was determined through title and abstract screening, followed by full-text screening of eligible articles. Data were extracted from the included studies to obtain quantitative outcomes, and a quality assessment was performed to assess the risk of bias. No review protocol was made, and the systematic review was not registered.

### Literature search

A systematic literature search was conducted to identify studies addressing urological cancer surveillance in individuals with LS. The search strategy comprised three categories: Lynch syndrome, urological cancer, and surveillance, and were combined using the Boolean operator “AND” with multiple search terms within each category combined using “OR.” In addition to the general search terms “urological cancer” and “urothelial carcinoma”, different combinations of search terms addressing prostate carcinoma, bladder and upper tract urothelial carcinoma, urethral carcinoma, renal cell carcinoma, germ cell tumors of the testis and penile carcinoma were used.

The MEDLINE database was searched via PubMed (https://pubmed.ncbi.nlm.nih.gov). Medical Subject Headings (MeSH) were applied, selecting the highest-level MeSH terms within the hierarchy tree with automatic explosion enabled. These terms were combined with title and abstract keyword searches to capture recently published articles not yet indexed with MeSH terms. To account for relevant phrases not included in the phrase index, proximity searches were performed within the title and abstract fields. The complete search string is provided in Supplementary Table 1. The initial search was conducted on September 4, 2025, and updated on November 6, 2025, to identify studies published in the interim. Additionally, reference lists of included studies were screened to identify further eligible publications.

### Eligibility criteria

Original studies published in English were eligible if they included a population of individuals with LS. Studies were required to report original data on surveillance strategies for urological malignancies, including the surveillance test used and their corresponding outcomes.

Studies were excluded if they were published in a language other than English or if the publication type was inappropriate, including reviews, editorials, commentaries, or case reports. Studies were also excluded if the study design was unsuitable.

### Study screening and data extraction

Title and abstract screening were conducted using Rayyan software [19]. Blinded screening at the title and abstract level, as well as full text screening and data extraction, were performed independently by both authors. Disagreements regarding study inclusion or exclusion were resolved through discussion until consensus was reached.

The following data were extracted from eligible studies: study characteristics, participant characteristics, surveillance test, and the efficacy of the surveillance tests. In cases of uncertainty regarding potential overlap of study populations or ambiguity in reported data, the corresponding authors were contacted for clarification.

### Outcome data

The primary outcomes of interest were the studied population, the surveillance strategy used in terms of frequency and method, the number of analyses performed for each method, the results of these analyses, and the number of cancers detected or missed by the surveillance strategy.

When more than one study reported results for the same surveillance test, a pooled analysis was performed to calculate sensitivity and specificity. When a study reported on more than one test, only the tests used as a surveillance test, and not follow-up examinations, were included in the data extraction.

### Quality assessment

The methodological quality of all included studies was independently assessed by both authors using the Newcastle–Ottawa Scale (NOS) [20]. Outcome of the quality assessment was used to estimate the risk of bias, but not to exclude publications, given the expected limited number of eligible studies. A truly representative cohort was considered as composed of LS individuals of all ages and MMR genes. If age criteria were set, or pathogenic variants missed, the cohort was considered somewhat representative. A selected group was defined when only one pathogenic variant or primarily one variant, or a high-risk LS group was included. A non-exposed cohort was defined as consisting of LS individuals without surveillance. Regarding the presence of the outcome, here PC or UC, as long as they were not selected due to having cancer, studies were awarded a star under “outcome not present at start”. When a statement regarding follow-up and individuals lost to follow-up was not given, bias assessment was based on the study design.

## Results

### Literature search and inclusion

**Fig. 1:**
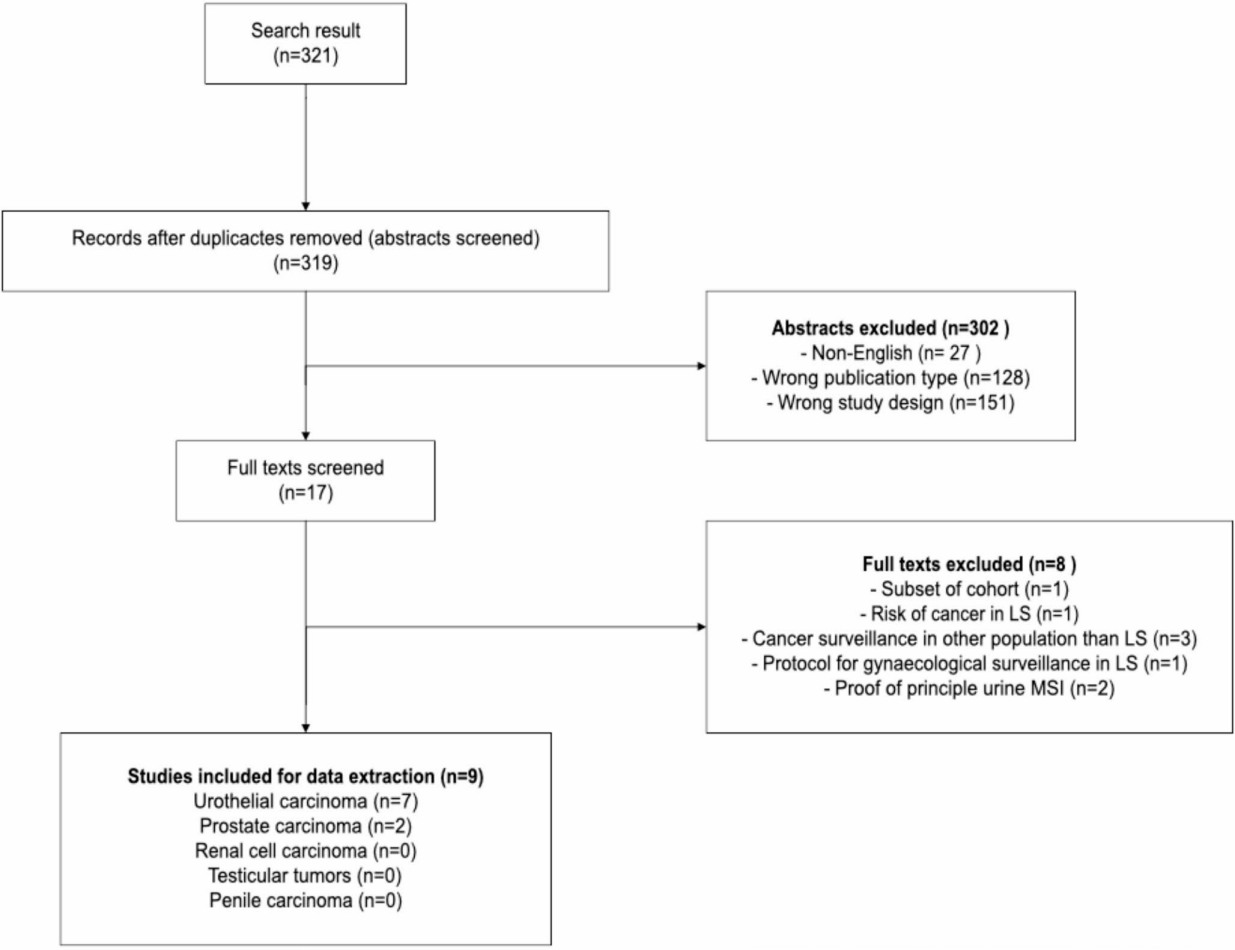
Search

The search string led to 312 possible hits. After removal of one duplicate, 311 abstracts were screened. The second search identified an additional 8 studies (and one duplicate) which were screened. The number of excluded studies and reason for exclusion are shown in Fig. 1.

Excluded publication types primarily comprised reviews and case reports (n = 128), inappropriate study design (n = 151), including those involving incorrect populations (predominantly patients with an established cancer diagnosis) or surveillance strategies not targeting urological cancer, among other reasons.

One study, by Kerr et al*.* [21] was excluded during full text screening because it reported on a subset of the cohort reported by Bancroft et al. [10], which was included being the larger and more recent cohort.

A total of nine studies [10, 15, 16, 22–27] were eligible for data extraction, including two studies on PC and seven studies on UC. No studies on renal cell carcinoma, testicular cancer or penile carcinoma were identified. The two PC studies originated from Norway and the United Kingdom, and the latter included LS individuals from eight different countries (Australia, Israel, Italy, Norway, Portugal, Spain, the United Kingdom, and the USA). The UC studies originated from five different countries (South Africa, Denmark, the Netherlands, the United Kingdom, and USA). Overall, publication years ranged from 2008 to 2025.

### Prostate carcinoma

The PC studies both had a prospective design, reporting on a total of 865 tested individuals with PSA tests from 2010 until 2020 (Table 1), which led to 38 PC diagnoses with 71% having a Gleason score > 6. Surveillance consisted of annual PSA tests from the age of 40 years in both studies. One study included all types of pathogenic MMR variants [22], while the other excluded individuals with a pathogenic *PMS2* variant because of a paucity of data supporting an increased risk of PC in this group [10].

**Table 1:** Included studies on prostate carcinoma

First author	Country of inclusion	Year	Study design	Single/multicenter	Years of inclusion	Age	Pathogenic variant	PSA threshold (ng/mL)*	Individuals tested (n)	Cancers detected (n)
Grindedal [22]	Norway	2024	Prospective	Single center	2010–2020	60.2 (mean, range 41–82)	MLH1 33 MSH2 69 MSH6 61 PMS 2 62	1995–1998: 3.4 1999–2004: 2.9 > 2005: 2.5	225	21
Bancroft [10]	Australia, Israel, Italy, Norway, Portugal, Spain, UK, USA	2021	Prospective/cross-sectional	Multi center	2012–2020	Median and IQR *MHL1*: 52 (46–59) *MSH2*: 51 (45–59) *MSH6*: 54 (46–60) *PMS2*: not included	MLH1 203 MSH2 303 MSH6 134 PMS2 not included	3.0	640	17

^*^Thresholds were changed during the study period

Bancroft et al. reported on baseline findings with 17 surveillance detected cancers in 644 tested individuals. One off protocol diagnosis was reported in a patient with a PSA of 0.85 ng/mL, which was below the screening threshold of 3.0 ng/nL, and was identified because of an abnormal digital rectal examination [10]. This results in a sensitivity and specificity of 94% and 96%, respectively. Grindedal et al. reported on surveillance over the course of 10 years with PC detected in 8 individuals at the first surveillance session and 14 cases found in one of the subsequent surveillance sessions. One PC was detected as a coincidental finding in a cystoprostatectomy specimen after UC treatment where the patient had a PSA value below the threshold one year before surgery (0.93 ng/mL). The remaining cases were found because of increased PSA levels [22].

### Urothelial carcinoma

A total of 1564 individuals underwent UC surveillance from 1991 until 2023 in which 11 individuals were diagnosed with UC and 15 missed diagnoses were reported on (Table 2). The surveillance strategies reported on were urinalysis, urine cytology, urine MSI, ultrasound and a combination of CT and cystoscopy with cytology.

**Table 2:** Studies on urothelial carcinoma

First author	Country of inclusion	Year	Study design	Single/multicenter	Years of inclusion	Age (mean and standard deviation (SD)/median and range)	Initial surveillance test	Individuals tested (n)	Gender (n, %)	Pathogenic variant (N, %)	Cancers detected with surveillance (n)	Cancers missed with surveillance (n)	Time since last surveillance to diagnosis (months)
Chouhan [27]	United States of America	2022	Retrospective	Single center	2008–2017	50 (20–74)	Urinalysis	204	Male: 102 (50) Female: 102 (50)	MLH1: 65 (32) MSH2: 93 (46) MSH6: 24 (12) PMS2: 22 (11)	0	5	1, 3, 6, 9, and 24
DeJesse [23]	United States of America	2021	Prospective	Single center	2017–2020	n/a	Urinalysis or urine cytology	99	n/a	n/a	1	Not mentioned	n/a
Doornweerd [26]	The Netherlands	2025	Retrospective	Multicenter	1992–2022	57 (24–90)	Urinalysis, urine cytology or ultrasound	95	n/a	n/a	0	2	< 12
Hall [15]	United Kingdom	2025	Prospective	Multicenter	2022–2023	57 (32–75)	Urine MSI, urinalysis, and ultrasound	80^*^	n/a	MSH2: 80 (100)	4	1 (missed by hematuria but identified with MSI)	n/a
Myrhøj [25]	Denmark	2008	Retrospective	Multicenter	1991–2005	47 (25–84)	Urine cytology	977**	Male: 451 (46) Female: 526 (54)	n/a	2	5	4–36
Pluke [24]	South Africa	2021	Cross-sectional	Multicenter	n/a	46 (SD 10.86)	Urinalysis	89	Male: 33 (37) Female: 56 (63)	MLH1: 86 (97) MSH2: 2 (2) Unknown: 1 (1)	0	Not mentioned	n/a
Zachhau [16]	Denmark	2012	Prospective	Single center	From 2001	n/a	CT and cystoscopy with immediate lavage cytology	20***	n/a	n/a	2	Not mentioned	n/a

^*^ Excluded one individual that developed UC prior to urine sample being made

^**^ Both LS and familial risk individuals

^***^ LS individuals with a family history of UC

### Urinalysis

Five studies reported on urinalysis results [15, 23, 24, 26, 27] (Table 2). In 490 Individuals, 631 urinalyses were performed from 1992 until 2023. In 1 individual UC was detected because of a positive test result [15], while 8 individuals with UC had negative urinalysis and were diagnosed because of symptoms (n = 5 [27]) or an abnormal MSI analysis of urine (N = 3 [15]) within 1–24 months after urinalysis (Table 2). In 62 cases abnormal urinalysis did not lead to the diagnosis of UC, thus considered false positives. A sensitivity of 11% and specificity of 90% were calculated from the reported data (Table 3).

**Table 3:** Test characteristics of different tests

Study (first author)	Individuals tested (n)	Number of tests (n)	True positive results (n)	False positive results (n)	True negative results (n)	False negative Results (n)	Sensitivity (%)	Specificity (%)
*Urinalysis*								
Chouhan [27]	204	n/a*	0	19	180	5	0	90
DeJesse [23]	99	197	0	9	188	0	n/a	95
Pluke [24]	89	89**	0	16	73	0	n/a	82
Hall [15]	55	55	1	3	48	3	25	94
Doornweerd [26]	43	86	0	15	71	0	n/a	83
Total urinalysis	490	631	1	62	560	8	11	90
*Cytology*								
DeJesse [23]	99	106	1	9	96	0	100	91
Myrhøj [25]	977	1868	2	36	1825	5	29	98
Doornweerd [26]	79	364***	0	19	343	2	0	95
Total cytology	1155	2338	3	64	2264	7	30	97
*Ultrasound*								
Hall [15]	44	44	0	9	34	1	0	79
Doornweerd [26]	50	105****	0	2	99	0	0	98
Total	94	149	0	11	133	1	0	92
*MSI*								
Hall [15]	80*****	80	4	1	75	0	100	99
*CT and cystoscopy*								
Zachhau [16]	20	26/48	2/1 ******	0	24/47	0	100/100	100/100

^*^Total number of tests was not reported, known results are used in this calculation

^**^Results were missing for 3 analyses, and removed from the data reported in this table

^***^Results were missing for 9 analyses, and removed from the data reported in this table

^****^Results were missing for 4 analyses, and removed from the data reported in this table

^*****^1 individual with hematuria was excluded

^******^1 distal ureter tumor was detected with both CT and cystoscopy

### Urine cytology

Three studies reported on urine cytology results [23, 25, 26] (Table 2). A fourth study reported on cytology as an adjunct to cystoscopy but did not report the complete results of all the tests and therefore were not included in the pooled analysis [16]. In 1171 individuals, 2328 cytologic examination of the urine were performed between 1991 until 2022. In 3 individuals an abnormal result led to a UC diagnosis, whereas 7 individuals with UC had a negative cytology result and were diagnosed because of symptoms 4–36 months after cytology [25, 26]. A sensitivity and specificity of 30% and 97%, respectively, were calculated (Table 3).

### Urine MSI

The study of Hall et al. [15] was the only included study to report on MSI testing of the urine and compared the results to existing methods: urinalysis and ultrasound. This study prospectively invited patients aged 30–75 to participate and performed 81 MSI tests in 81 eligible individuals from 2022 until 2023. One individual developed hematuria because of UC prior to MSI analysis, and this patient was not included in the data extracted although urine MSI was positive. Additionally, 4 cases of UC were detected with urine MSI and 1 test was false positive, thus 100% sensitivity and 99% specificity were reached (Table 3).

### Ultrasound

Two studies reported on abdominal ultrasound [15, 26] (Table 2). In 94 individuals, 149 ultrasounds were performed from 1992 until 2023. Of these, 11 examinations showed benign abnormalities. None of the ultrasounds detected a UC, however 1 showed a renal mass while missing a bladder cancer [15, 26]. This gave an overall sensitivity of 0% and a specificity of 92% (Table 3).

### CT and cystoscopy

Zachhau and Walter reported on CT and cystoscopy with cytology in 20 individuals with LS and a positive family history for UC [16] (Table 2). In here, 2 patients with upper urinary UC were identified because of an abnormal CT, and no cancer was missed with 48 performed cystoscopies with cytology and 26 performed CT’s leading to both 100% sensitivity and specificity (Table 3).

### Individuals tested

Characteristics of the tested individuals vary among the included studies. Age ranged from 20 to 90 years old. Of the two prospective cohort studies Hall et al*.* [15] invited individuals from 30 to 75 years old, whereas Zachhau and Walter [16] did not report on the age of invited individuals. All other studies are registry studies and reported on median or mean age. In three studies [23, 26, 27] individuals with all pathogenic MMR genes were included, whereas one study, by Hall et al*.* selected only individuals with a pathogenic *MSH2* variant [15]. Pluke and Kaestner reported 98% *MLH1* carriers and 2% *MSH2* carriers [24]. Myrhøj et al. selected families with a proven pathogenic variant, as well as families meeting the Amsterdam I or II criteria and families with a suspicion of LS, but the MMR variants were not reported on [25]. Lastly, Zachhau and Walter selected individuals with LS and two family members with UC but did not specify MMR genes [16].

### Recommended surveillance program

The frequency of surveillance tests reported on was not specified in two studies [23, 26], whereas two studies reported on single outcomes [15, 24]. Chouhan et al*.* reported on annual testing using cytology starting at age 35 [27], Myrhøj et al*.* recommended biennial cytology from age 25 [25], and Zachhau and Walter reported on biennial surveillance using CT and cystoscopy with cytology for LS individuals with a family history of UC [16].

### Quality assessment

Most studies on UC were retrospective observational cohort studies. Overall, the majority of the nine studies did not report on a cohort of individuals with LS that did not undergo surveillance for UC, defined as a non-exposed cohort by the NOS, and comparison between cohorts was not possible or done. Results of quality assessment are shown in supplementary Table 2. Overall, the score ranged from 5 to 7 stars.

## Discussion

While there is a high risk of urological malignancies for LS individuals and a high mortality, there is no consensus on urological surveillance in LS individuals. The literature search identified nine cohort studies with original data on urological surveillance in LS individuals. Two studies reported on surveillance for PC using annual PSA as the method, with a sensitivity of 94% and specificity of 96% based on the study by Bancroft et al. For UC, seven studies reported on surveillance with urinalysis, urine cytology, urine MSI, ultrasound and/or CT and cystoscopy with cytology as the methods. Urinalysis had an overall sensitivity and specificity of 11% and 90%, respectively. For cytology the sensitivity was 30% and the specificity was 97%, while it for urine MSI analysis was 100% and 97%, respectively. For ultrasound there was a sensitivity of 0% and a specificity of 92%. Lastly, for CT and cystoscopy combined with cytology, the sensitivity and specificity were both 100%. No studies on renal cell carcinoma, testicular cancer, or penile cancer were found.

### Prostate carcinoma

An increased risk for PC in LS is reported on in both studies included, but histology specimens weren’t tested for MMR protein deficiency or MSI. Given the high prevalence of PC in the general population sporadic cases in the individuals with LS cannot be ruled out. Both studies had an initiation age of 40 years and a surveillance interval of 1 year. This is in contrast to the ERSPC, a large population-based PC screening study, which started at age 55 and reported intervals between 2 and 7 years [28]. Given the observed PC diagnosis at a younger age in the PLSD database [2] and the high proportion of aggressive PC in LS reported on in the studies included in this review, this early start and high frequency may be justified.

Bancroft et al. [10] described one missed diagnosis in a patient with a PSA level below the screening threshold; however, follow-up data were insufficient to identify additional missed cases. It should be taken into consideration that the sensitivity can change when longer follow-up data become available [10]. Grindedal et al. reported longer follow-up of up to 10 years but as the total number of tests was not described, sensitivity and specificity could not be calculated [22]. However, they also reported a missed cancer with a PSA value below the threshold. The PSA threshold was lowered during the study period, but an exact threshold needs to be researched to determine optimal sensitivity and specificity for a surveillance program [22]. Altogether these findings support a PC surveillance program in individuals with LS. However, whether such a program translates into improved patient outcomes and how the exact program should be designed, remain to be determined.

### Urothelial carcinoma

#### CT and cystoscopy

While the best sensitivity was reached for CT and cystoscopy combined with cytology, this method was only used for a small number of individuals with LS that had a family history of UC and were deemed at high risk of developing UC. An important limitation of this strategy is radiation exposure. When CT is used to replace an invasive procedure in a low frequency, the advantages may outweigh the disadvantages, but when used frequently the radiation may have more negative than positive health implications [29]. For a successful UC surveillance program in LS, CT’s should be performed from a young age and with a high frequency, which could lead to considerable negative health effects. In addition to the risk of radiation, intravenous contrast, used for CT urography, can lead to contrast nephropathy. Although the effects of intravenous contrast on kidney function are controversial [30], it should be used with caution in individuals who may need cancer treatment that impairs kidney function, for example nephrectomy in the case of UC of the renal pelvis. In addition to the limitations of CT, cystoscopy has some inherent limitations as well. Cystoscopy (in combination with cytology or not), being an endoscopic examination of the lower urinary tract, can detect cancer in the bladder but is not appropriate for detecting UC of the upper urinary tract [17]. Furthermore, it is invasive and generally considered an uncomfortable examination [31]. This can potentially affect the compliance to surveillance for these individuals. Lastly, when this strategy is used in the surveillance of treated UC, it has shown to be very expensive [32].

Despite the good sensitivity for finding UC, due to possible adverse effects, invasiveness and costs, CT and cystoscopy should be reserved as complementary procedures in individuals with abnormal surveillance tests to confirm a UC diagnosis.

### Ultrasound

In contrast to CT, a non-invasive method such as ultrasound is, as demonstrated by the (limited) results in this review, not an effective method for UC surveillance, which is in line with the decreasing use of ultrasound in upper urinary tract imaging [33].

### Urinalysis

Most included UC studies reported a low sensitivity for urinalysis as a surveillance test for UC. Sensitivity is dependent on the threshold set for a positive test, and changes when changing the threshold [34, 35]. In this review the threshold was not taken into consideration, because of the limited availability of these data. A large meta-analysis evaluating testing for asymptomatic non-visible hematuria in unselected populations demonstrated a low positive predictive value for the detection of urothelial and kidney cancer. The reason for low sensitivity can be explained by red blood cell lysis, intermittent hematuria and uncertain thresholds, among other things [36]. In the included studies, cancers missed by urinalysis were mostly found because of symptoms. The study by Hall et al. [15] identified one individual with UC with urinalysis, but in this case MSI analysis was abnormal as well. The remaining three cancers identified via MSI were missed by urinalysis. In addition to the low sensitivity, true positive test results in the included studies were outnumbered by a high number of false positives (62 of 631 tests) leading to unnecessary examinations and possible distress. The evidence remains scarce, but urinalysis has considerable limitations as a surveillance test.

### Urine cytology

A pooled analysis of urine cytology data in this review demonstrated a sensitivity of 30%, which is lower than when used in symptomatic individuals with hematuria [37]. The largest study in this review, by Myrhøj et al., reported on missed cancers with an interval of 4–36 months. The shorter interval could be considered a false-negative while the longer interval is unlikely to represent a true false-negative surveillance result given the short lead time of UC [25]. The latter might have been identified if cytology surveillance had been done more frequently. DeJesse et al. reported 100% sensitivity, but their study lacked sufficient statistical power to allow firm conclusions [23]. Doornweerd et al. reported a small number of false negative cytology results with no true-positives, leading to a sensitivity of 0%, but this is questionable given the small numbers [26]. The observed range in sensitivity of 0–100% highlights the need for a large (international) cohort to determine the true sensitivity of cytology in LS. Being historical databases, sensitivity of urine cytology may be low because of reporting discrepancies due to changing guidelines. When reported according to the newest “The Paris System” (TPS 2.0), cytology may reach a sensitivity of up to 70% for high grade UC. Sensitivity for low grade UC is much lower and the newest TPS version is moving from a ‘risk of malignancy’ assessment to a ‘risk of high-grade malignancy’ assessment [38]. Given the risk for UC in LS and the need for organ-sparing surgery, if possible, it could be argued that surveillance should not be aimed at high grade UC solely, disqualifying urine cytology as a proper surveillance method in LS.

### Urine MSI

Urine MSI analysis reached a sensitivity of 100%, making it the most effective non-invasive surveillance option without radiation exposure reported in this review, approaching the sensitivity and specificity found for CT and cystoscopy combined. Although the small number of individuals and the selection of individuals with *MSH2* make the data unsuitable for conclusions on all individuals with LS, the results are promising [15]. Larger studies with more cancer cases and analysis of individuals with all pathogenic variants are required to validate these findings. Furthermore, while the other methods are more established methods, this is a newer assay, and it would need proper implementation to be used as a standardized surveillance method.

### Surveillance protocol

The composition of the included cohorts varies greatly, with one study only including individuals with a pathogenic *MSH2* variant, while others included all MMR genes. In one study also individuals suspected of a familiar risk were included. Selection of *MSH2* seems justifiable as the cumulative incidence is highest for these individuals, but little is known about the biological behavior of UC caused by the different MMR genes. Withholding surveillance to individuals with other pathogenic variants than *MSH2* should therefore be done cautiously. Furthermore, the age to start surveillance cannot be concluded from this review and should be the topic of further research. As most of the included studies were done retrospectively, the data on frequency of the performed surveillance is scarce. A possible survival benefit of shorter waiting times for treatment and favorable outcome of asymptomatic patients with UC compared to symptomatic patients support more frequent testing [39, 40], although there is no available evidence that urologic surveillance impacts outcomes in urologic cancers in LS. For now, it is assumed that an early diagnosis leads to better treatment perspectives. The most effective surveillance interval still needs to be determined in relation to a proposed surveillance method.

### Limitations

An important limitation of this review is the scarcity of available original data. Although a substantial number of reviews have been published, the body of original research addressing urological surveillance in individuals with LS is limited to only 9 studies. Some methods only were tested in a small cohort of LS individuals making interpretation difficult. Furthermore, not all included individuals were confirmed to have LS and not all pathogenic variants were analyzed for all methods, which limits the possibilities of robust conclusions. The largest cohort reporting on the use of urine cytology included individuals without a proven pathogenic variant. Therefore, the reported UC diagnoses in this cohort could be a wrong estimation of the truly LS associated UC’s [25].

### Possible bias

Quality assessment using the NOS indicated bias in some of the included studies. Although the NOS is a commonly used instrument for assessing methodological quality, its validity has been questioned [41]. For most studies included in this review, the applicability of the NOS was limited due to their study designs. This was mainly due to a more selected cohort, no presence of a non-exposed cohort, such as individuals not undergoing surveillance, and therefore also no comparison between the groups. None of the studies used a reference standard in the individuals with a negative surveillance test, which might have led to an underestimation of false negative results. Furthermore, none prospectively compared an unselected cohort of LS individuals that underwent surveillance, to an equivalent non-exposed control cohort, during a longer period. For the aim of this review the quality was deemed acceptable.

### Further research

In this review, surveillance test performance, including sensitivity and specificity, was taken into consideration, but for a test to be implemented, multiple factors need to be considered.

Preferably a surveillance test should be a test that is easily performed, is non-invasive, has low costs and is generally available, without compromising sensitivity or specificity. It should be determined which LS individuals should undergo urological surveillance, given the different risk for UC in the different pathogenic MMR gene variants. The questions at what age urological surveillance should start and the interval between surveillance sessions still need to be answered as well. Most important, the question if UC surveillance leads to better treatment outcome and survival in LS needs to be answered. Finally, the acceptance of the tests by LS individuals and willingness to attend surveillance needs to be addressed.

## Conclusion

For PC surveillance, PSA shows promise, while for UC urine MSI analysis shows promise for *MSH2* carriers, at least. However, data on urological cancer surveillance for LS individuals is scarce and more research is needed to recommend a specific surveillance program.

## Supplementary Information

Below is the link to the electronic supplementary material.

Supplementary Material 1

Supplementary Material 2

## Data Availability

No datasets were generated or analysed during the current study.
