# Supplementary material for: Early detection of urological malignancies in Lynch syndrome: a systematic review
Source: Fam Cancer. 2026 Apr 24;25(2):45. doi: 10.1007/s10689-026-00560-5 (PMC13109156; doi:10.1007/s10689-026-00560-5)
Supplement: Supplementary file 1 — Supplementary Material 1 [file 10689_2026_560_MOESM1_ESM.docx]

| Supplementary table 2. Newcastle-Ottawa quality assessment for the included studies | | | | | | | | | |
| --- | --- | --- | --- | --- | --- | --- | --- | --- | --- |
| Study | Selection | | | | Comparability | Outcome | | | Overall risk of bias |
|  | Representativeness | Selection of non-exposed cohort | Ascertainment of exposure | Outcome not present at start | Controlled for confounders | Assessment | Follow-up length sufficient | Adequacy of follow-up | Total number of stars |
| Bancroft | *Somewhat representative* | No description/not available | *Secure record* | *Yes* | No cohort without surveillance | *Record linkage* | No | *Assumed complete based on study design* | 6 |
| Grindedal | *Somewhat representative* | No description/not available | *Secure record* | *Yes* | No cohort without surveillance | *Record linkage* | *Yes* | *Assumed complete based on study design* | 6 |
| Chouhan | *Truly representative* | No description/not available | *Secure record* | *Yes* | Not comparable/not relevant | *Record linkage* | *Yes* | *Assumed complete based on study design* | 6 |
| DeJesse | *Truly representative* | *Same community* | *Secure record* | *Yes* | Not comparable/controls for only a subset of included individuals | *Record linkage* | No/nothing mentioned | *Assumed complete based on study design* | 6 |
| Doornweerd | *Truly representative* | *Same community* | *Secure record* | *Yes* | Not comparable | *Record linkage* | *Yes* | *Unlikely to introduce bias* | 7 |
| Hall | Selected group | No description/not available | *Secure record* | *Yes* | No cohort without surveillance | *Record linkage* | *Yes* | *Complete* | 5 |
| Myrhøj | *Somewhat representative* | No description/not available | *Secure record* | *Yes* | No cohort without surveillance | *Record linkage* | *Yes* | *Assumed complete based on study design* | 6 |
| Pluke and Kaestner | Selected group | No description/not available | *Secure record* | *Yes* | No cohort without surveillance | *Record linkage* | No | *Assumed complete based on study design* | 5 |
| Zachhau and Walter | Selected group | No description/not available | *Secure record* | *Yes* | No cohort without surveillance | *Record linkage* | *Yes* | *Unlikely to introduce bias* | 5 |
| Items awarded a star are marked italic | | | | | | | | | |
