# Supplementary material for: Early detection of urological malignancies in Lynch syndrome: a systematic review
Source: Fam Cancer. 2026 Apr 24;25(2):45. doi: 10.1007/s10689-026-00560-5 (PMC13109156; doi:10.1007/s10689-026-00560-5)
Supplement: Supplementary file 2 — Supplementary Material 2 [file 10689_2026_560_MOESM2_ESM.docx]

| Supplementary table 1: Search string |
| --- |
| ("colorectal neoplasms, hereditary nonpolyposis"[MeSH Terms] OR "Lynch"[Title/Abstract] OR "Hereditary Nonpolyposis Colon Cancer"[Title/Abstract] OR "Mismatch repair"[Title/Abstract] OR "Microsatellite instability"[Title/Abstract] OR "Hereditary Nonpolyposis Colorectal Cancer"[Title/Abstract]) AND ("Kidney cancer"[Title/Abstract:~0] OR "Kidney cancers"[Title/Abstract:~0] OR "Kidney carcinoma"[Title/Abstract:~0] OR "Kidney carcinomas"[Title/Abstract:~0] OR "Kidney neoplasm"[Title/Abstract:~0] OR "Kidney neoplasms"[Title/Abstract:~0] OR "Kidney tumor"[Title/Abstract:~0] OR "Kidney tumors"[Title/Abstract:~0] OR "Kidney tumour"[Title/Abstract:~0] OR "Kidney tumours"[Title/Abstract:~0] OR "Kidney malignancy"[Title/Abstract:~0] OR "Kidney malignancies"[Title/Abstract:~0] OR "Renal cell carcinoma"[Title/Abstract:~0] OR "Renal cell carcinomas"[Title/Abstract:~0] OR "Renal cell cancer"[Title/Abstract:~0] OR "Renal cell cancers"[Title/Abstract:~0] OR "Renal cell neoplasm"[Title/Abstract:~0] OR "Renal cell neoplasms"[Title/Abstract:~0] OR "Renal cell tumor"[Title/Abstract:~0] OR "Renal cell tumors"[Title/Abstract:~0] OR "Renal cell tumour"[Title/Abstract:~0] OR "Renal cell tumours"[Title/Abstract:~0] OR "Renal cell malignancy"[Title/Abstract:~0] OR "Renal cell malignancies"[Title/Abstract:~0] OR "Renal carcinoma"[Title/Abstract:~0] OR "Renal carcinomas"[Title/Abstract:~0] OR "Renal cancer"[Title/Abstract:~0] OR "Renal cancers"[Title/Abstract:~0] OR "Renal neoplasm"[Title/Abstract:~0] OR "Renal neoplasms"[Title/Abstract:~0] OR "Renal tumor"[Title/Abstract:~0] OR "Renal tumors"[Title/Abstract:~0] OR "Renal tumour"[Title/Abstract:~0] OR "Renal tumours"[Title/Abstract:~0] OR "Renal malignancy"[Title/Abstract:~0] OR "Renal malignancies"[Title/Abstract:~0] OR "Renal pelvis carcinoma"[Title/Abstract:~0] OR "Renal pelvis carcinomas"[Title/Abstract:~0] OR "Renal pelvis cancer"[Title/Abstract:~0] OR "Renal pelvis cancers"[Title/Abstract:~0] OR "Renal pelvis neoplasm"[Title/Abstract:~0] OR "Renal pelvis neoplasms"[Title/Abstract:~0] OR "Renal pelvis tumor"[Title/Abstract:~0] OR "Renal pelvis tumors"[Title/Abstract:~0] OR "Renal pelvis tumour"[Title/Abstract:~0] OR "Renal pelvis tumours"[Title/Abstract:~0] OR "Renal pelvis malignancy"[Title/Abstract:~0] OR "Renal pelvis malignancies"[Title/Abstract:~0] OR "Renal pelvic carcinoma"[Title/Abstract:~0] OR "Renal pelvic carcinomas"[Title/Abstract:~0] OR "Renal pelvic cancer"[Title/Abstract:~0] OR "Renal pelvic cancers"[Title/Abstract:~0] OR "Renal pelvic neoplasm"[Title/Abstract:~0] OR "Renal pelvic neoplasms"[Title/Abstract:~0] OR "Renal pelvic tumor"[Title/Abstract:~0] OR "Renal pelvic tumors"[Title/Abstract:~0] OR "Renal pelvic tumour"[Title/Abstract:~0] OR "Renal pelvic tumours"[Title/Abstract:~0] OR "Renal pelvic malignancy"[Title/Abstract:~0] OR "Renal pelvic malignancies"[Title/Abstract:~0] OR "Renal pelvis urothelial carcinoma"[Title/Abstract:~0] OR "Renal pelvis urothelial carcinomas"[Title/Abstract:~0] OR "Renal pelvis urothelial cancer"[Title/Abstract:~0] OR "Renal pelvis urothelial cancers"[Title/Abstract:~0] OR "Renal pelvis urothelial neoplasm"[Title/Abstract:~0] OR "Renal pelvis urothelial neoplasms"[Title/Abstract:~0] OR "Renal pelvis urothelial tumor"[Title/Abstract:~0] OR "Renal pelvis urothelial tumors"[Title/Abstract:~0] OR "Renal pelvis urothelial tumour"[Title/Abstract:~0] OR "Renal pelvis urothelial tumours"[Title/Abstract:~0] OR "Renal pelvis malignancy"[Title/Abstract:~0] OR "Renal pelvis malignancies"[Title/Abstract:~0] OR "Renal pelvic urothelial carcinoma"[Title/Abstract:~0] OR "Renal pelvic urothelial carcinomas"[Title/Abstract:~0] OR "Renal pelvic urothelial cancer"[Title/Abstract:~0] OR "Renal pelvic urothelial cancers"[Title/Abstract:~0] OR "Renal pelvic neoplasm"[Title/Abstract:~0] OR "Renal pelvic urothelial neoplasms"[Title/Abstract:~0] OR "Renal pelvic urothelial tumor"[Title/Abstract:~0] OR "Renal pelvic urothelial tumors"[Title/Abstract:~0] OR "Renal pelvic urothelial tumour"[Title/Abstract:~0] OR "Renal pelvic urothelial tumours"[Title/Abstract:~0] OR "Renal pelvic urothelial malignancy"[Title/Abstract:~0] OR "Renal pelvic urothelial malignancies"[Title/Abstract:~0] OR "Ureter carcinoma"[Title/Abstract:~0] OR "Ureter carcinomas"[Title/Abstract:~0] OR "Ureter cancer"[Title/Abstract:~0] OR "Ureter cancers"[Title/Abstract:~0] OR "Ureter neoplasm"[Title/Abstract:~0] OR "Ureter neoplasms"[Title/Abstract:~0] OR "Ureter tumor"[Title/Abstract:~0] OR "Ureter tumors"[Title/Abstract:~0] OR "Ureter tumour"[Title/Abstract:~0] OR "Ureter tumours"[Title/Abstract:~0] OR "Ureter malignancy"[Title/Abstract:~0] OR "Ureter malignancies"[Title/Abstract:~0] OR "Ureteral carcinoma"[Title/Abstract:~0] OR "Ureteral carcinomas"[Title/Abstract:~0] OR "Ureteral cancer"[Title/Abstract:~0] OR "Ureteral cancers"[Title/Abstract:~0] OR "Ureteral neoplasm"[Title/Abstract:~0] OR "Ureteral neoplasms"[Title/Abstract:~0] OR "Ureteral tumor"[Title/Abstract:~0] OR "Ureteral tumors"[Title/Abstract:~0] OR "Ureteral tumour"[Title/Abstract:~0] OR "Ureteral tumours"[Title/Abstract:~0] OR "Ureteral malignancy"[Title/Abstract:~0] OR "Ureteral malignancies"[Title/Abstract:~0] OR "Ureteric carcinoma"[Title/Abstract:~0] OR "Ureteric carcinomas"[Title/Abstract:~0] OR "Ureteric cancer"[Title/Abstract:~0] OR "Ureteric cancers"[Title/Abstract:~0] OR "Ureteric neoplasm"[Title/Abstract:~0] OR "Ureteric neoplasms"[Title/Abstract:~0] OR "Ureteric tumor"[Title/Abstract:~0] OR "Ureteric tumors"[Title/Abstract:~0] OR "Ureteric tumour"[Title/Abstract:~0] OR "Ureteric tumours"[Title/Abstract:~0] OR "Ureteric malignancy"[Title/Abstract:~0] OR "Ureteric malignancies"[Title/Abstract:~0] OR "Urethra carcinoma"[Title/Abstract:~0] OR "Urethra carcinomas"[Title/Abstract:~0] OR "Urethra cancer"[Title/Abstract:~0] OR "Urethra cancers"[Title/Abstract:~0] OR "Urethra neoplasm"[Title/Abstract:~0] OR "Urethra neoplasms"[Title/Abstract:~0] OR "Urethra tumor"[Title/Abstract:~0] OR "Urethra tumors"[Title/Abstract:~0] OR "Urethra tumour"[Title/Abstract:~0] OR "Urethra tumours"[Title/Abstract:~0] OR "Urethra malignancy"[Title/Abstract:~0] OR "Urethra malignancies"[Title/Abstract:~0] OR "Urethral carcinoma"[Title/Abstract:~0] OR "Urethral carcinomas"[Title/Abstract:~0] OR "Urethral cancer"[Title/Abstract:~0] OR "Urethral cancers"[Title/Abstract:~0] OR "Urethral neoplasm"[Title/Abstract:~0] OR "Urethral neoplasms"[Title/Abstract:~0] OR "Urethral tumor"[Title/Abstract:~0] OR "Urethral tumors"[Title/Abstract:~0] OR "Urethral tumour"[Title/Abstract:~0] OR "Urethral tumours"[Title/Abstract:~0] OR "Urethral malignancy"[Title/Abstract:~0] OR "Urethral malignancies"[Title/Abstract:~0] OR "Bladder carcinoma"[Title/Abstract:~0] OR "Bladder carcinomas"[Title/Abstract:~0] OR "Bladder cancer"[Title/Abstract:~0] OR "Bladder cancers"[Title/Abstract:~0] OR "Bladder neoplasm"[Title/Abstract:~0] OR "Bladder neoplasms"[Title/Abstract:~0] OR "Bladder tumor"[Title/Abstract:~0] OR "Bladder tumors"[Title/Abstract:~0] OR "Bladder tumour"[Title/Abstract:~0] OR "Bladder tumours"[Title/Abstract:~0] OR "Bladder malignancy"[Title/Abstract:~0] OR "Bladder malignancies"[Title/Abstract:~0] OR "Urothelial carcinoma"[Title/Abstract:~0] OR "Urothelial carcinomas"[Title/Abstract:~0] OR "Urothelial cancer"[Title/Abstract:~0] OR "Urothelial cancers"[Title/Abstract:~0] OR "Urothelial neoplasm"[Title/Abstract:~0] OR "Urothelial neoplasms"[Title/Abstract:~0] OR "Urothelial tumor"[Title/Abstract:~0] OR "Urothelial tumors"[Title/Abstract:~0] OR "Urothelial tumour"[Title/Abstract:~0] OR "Urothelial tumours"[Title/Abstract:~0] OR "Urothelial malignancy"[Title/Abstract:~0] OR "Urothelial malignancies"[Title/Abstract:~0] OR "Urothelial cell carcinoma"[Title/Abstract:~0] OR "Urothelial cell carcinomas"[Title/Abstract:~0] OR "Urothelial cell cancer"[Title/Abstract:~0] OR "Urothelial cell cancers"[Title/Abstract:~0] OR "Urothelial cell neoplasm"[Title/Abstract:~0] OR "Urothelial cell neoplasms"[Title/Abstract:~0] OR "Urothelial cell tumor"[Title/Abstract:~0] OR "Urothelial cell tumors"[Title/Abstract:~0] OR "Urothelial cell tumour"[Title/Abstract:~0] OR "Urothelial cell tumours"[Title/Abstract:~0] OR "Urothelial cell malignancy"[Title/Abstract:~0] OR "Urothelial cell malignancies"[Title/Abstract:~0] OR "Transitional cell carcinoma"[Title/Abstract:~0] OR "Transitional cell carcinomas"[Title/Abstract:~0] OR "Transitional cell cancer"[Title/Abstract:~0] OR "Transitional cell cancers"[Title/Abstract:~0] OR "Transitional cell neoplasm"[Title/Abstract:~0] OR "Transitional cell neoplasms"[Title/Abstract:~0] OR "Transitional cell tumor"[Title/Abstract:~0] OR "Transitional cell tumors"[Title/Abstract:~0] OR "Transitional cell tumour"[Title/Abstract:~0] OR "Transitional cell tumours"[Title/Abstract:~0] OR "Transitional cell malignancy"[Title/Abstract:~0] OR "Transitional cell malignancies"[Title/Abstract:~0] OR "Uroepithelial carcinoma"[Title/Abstract:~0] OR "Uroepithelial carcinomas"[Title/Abstract:~0] OR "Uroepithelial cancer"[Title/Abstract:~0] OR "Uroepithelial cancers"[Title/Abstract:~0] OR "Uroepithelial neoplasm"[Title/Abstract:~0] OR "Uroepithelial neoplasms"[Title/Abstract:~0] OR "Uroepithelial tumor"[Title/Abstract:~0] OR "Uroepithelial tumors"[Title/Abstract:~0] OR "Uroepithelial tumour"[Title/Abstract:~0] OR "Uroepithelial tumours"[Title/Abstract:~0] OR "Uroepithelial malignancy"[Title/Abstract:~0] OR "Uroepithelial malignancies"[Title/Abstract:~0] OR "Germ cell carcinoma"[Title/Abstract:~0] OR "Germ cell carcinomas"[Title/Abstract:~0] OR "Germ cell cancer"[Title/Abstract:~0] OR "Germ cell cancers"[Title/Abstract:~0] OR "Germ cell tumor"[Title/Abstract:~0] OR "Germ cell tumors"[Title/Abstract:~0] OR "Germ cell tumour"[Title/Abstract:~0] OR "Germ cell tumours"[Title/Abstract:~0] OR "Germ cell malignancy"[Title/Abstract:~0] OR "Germ cell malignancies"[Title/Abstract:~0] OR "Testis carcinoma"[Title/Abstract:~0] OR "Testis carcinomas"[Title/Abstract:~0] OR "Testis cancer"[Title/Abstract:~0] OR "Testis cancers"[Title/Abstract:~0] OR "Testis tumor"[Title/Abstract:~0] OR "Testis tumors"[Title/Abstract:~0] OR "Testis tumour"[Title/Abstract:~0] OR "Testis tumours"[Title/Abstract:~0] OR "Testis malignancy"[Title/Abstract:~0] OR "Testis malignancies"[Title/Abstract:~0] OR "Testicular carcinoma"[Title/Abstract:~0] OR "Testicular carcinomas"[Title/Abstract:~0] OR "Testicular cancer"[Title/Abstract:~0] OR "Testicular cancers"[Title/Abstract:~0] OR "Testicular tumor"[Title/Abstract:~0] OR "Testicular tumors"[Title/Abstract:~0] OR "Testicular tumour"[Title/Abstract:~0] OR "Testicular tumours"[Title/Abstract:~0] OR "Testicular malignancy"[Title/Abstract:~0] OR "Testicular malignancies"[Title/Abstract:~0] OR "Seminoma"[Title/Abstract] OR "Non-seminoma"[Title/Abstract:~0] OR "Embryonal cell carcinoma"[Title/Abstract:~0] OR "Yolk sac"[Title/Abstract:~0] OR "Teratoma"[Title/Abstract] OR "Prostate carcinoma"[Title/Abstract:~0] OR "Prostate carcinomas"[Title/Abstract:~0] OR "Prostate cancer"[Title/Abstract:~0] OR "Prostate cancers"[Title/Abstract:~0] OR "Prostate neoplasm"[Title/Abstract:~0] OR "Prostate neoplasms"[Title/Abstract:~0] OR "Prostate tumor"[Title/Abstract:~0] OR "Prostate tumors"[Title/Abstract:~0] OR "Prostate tumour"[Title/Abstract:~0] OR "Prostate tumours"[Title/Abstract:~0] OR "Prostate malignancy"[Title/Abstract:~0] OR "Prostate malignancies"[Title/Abstract:~0] OR "Prostatic carcinoma"[Title/Abstract:~0] OR "Prostatic carcinomas"[Title/Abstract:~0] OR "Prostatic cancer"[Title/Abstract:~0] OR "Prostatic cancers"[Title/Abstract:~0] OR "Prostatic neoplasm"[Title/Abstract:~0] OR "Prostatic neoplasms"[Title/Abstract:~0] OR "Prostatic tumor"[Title/Abstract:~0] OR "Prostatic tumors"[Title/Abstract:~0] OR "Prostatic tumour"[Title/Abstract:~0] OR "Prostatic tumours"[Title/Abstract:~0] OR "Prostatic malignancy"[Title/Abstract:~0] OR "Prostatic malignancies"[Title/Abstract:~0] OR "Urinary tract carcinoma"[Title/Abstract:~0] OR "Urinary tract carcinomas"[Title/Abstract:~0] OR "Urinary tract cancer"[Title/Abstract:~0] OR "Urinary tract cancers"[Title/Abstract:~0] OR "Urinary tract neoplasm"[Title/Abstract:~0] OR "Urinary tract neoplasms"[Title/Abstract:~0] OR "Urinary tract tumor"[Title/Abstract:~0] OR "Urinary tract tumors"[Title/Abstract:~0] OR "Urinary tract tumour"[Title/Abstract:~0] OR "Urinary tract tumours"[Title/Abstract:~0] OR "Urinary tract malignancy"[Title/Abstract:~0] OR "Urinary tract malignancies"[Title/Abstract:~0] OR "Penis carcinoma"[Title/Abstract:~0] OR "Penis carcinomas"[Title/Abstract:~0] OR "Penis cancer"[Title/Abstract:~0] OR "Penis cancers"[Title/Abstract:~0] OR "Penis neoplasm"[Title/Abstract:~0] OR "Penis neoplasms"[Title/Abstract:~0] OR "Penis tumor"[Title/Abstract:~0] OR "Penis tumors"[Title/Abstract:~0] OR "Penis tumour"[Title/Abstract:~0] OR "Penis tumours"[Title/Abstract:~0] OR "Penis malignancy"[Title/Abstract:~0] OR "Penis malignancies"[Title/Abstract:~0] OR "Penile carcinoma"[Title/Abstract:~0] OR "Penile carcinomas"[Title/Abstract:~0] OR "Penile cancer"[Title/Abstract:~0] OR "Penile cancers"[Title/Abstract:~0] OR "Penile neoplasm"[Title/Abstract:~0] OR "Penile neoplasms"[Title/Abstract:~0] OR "Penile tumor"[Title/Abstract:~0] OR "Penile tumors"[Title/Abstract:~0] OR "Penile tumour"[Title/Abstract:~0] OR "Penile tumours"[Title/Abstract:~0] OR "Penile malignancy"[Title/Abstract:~0] OR "Penile malignancies"[Title/Abstract:~0] OR "genital neoplasms, male"[MeSH Terms] OR "urologic neoplasms"[MeSH Terms]) AND ("Early detection of cancer"[MeSH Terms] OR "Prostate specific antigen"[MeSH Terms] OR "Screening"[Title/Abstract] OR "Surveillance"[Title/Abstract] OR "Early cancer detection"[Title/Abstract] OR "Early detection"[Title/Abstract] OR "Hematuria"[Title/Abstract] OR "Haematuria"[Title/Abstract] OR "Urine cytology"[Title/Abstract] OR "Urinary cytology"[Title/Abstract] OR "Ultrasound"[Title/Abstract] OR "PSA"[Title/Abstract] OR "Prostate specific antigen"[Title/Abstract]) |
